# Investigation of Roughness Correlation in Polymer Brushes via X-ray Scattering

**DOI:** 10.3390/polym12092101

**Published:** 2020-09-15

**Authors:** Marcus Hildebrandt, Eui-young Shin, Suan Yang, Wael Ali, Sedakat Altinpinar, Jochen S. Gutmann

**Affiliations:** 1 Department of Physical Chemistry and Center of Nanointegration (CENIDE), University of Duisburg-Essen, Universitätsstr. 2, 45141 Essen, Germany; Eui-young.shin@stud.uni-due.de (E.-y.S.); suan.yang@stud.uni-due.de (S.Y.); ali@dtnw.de (W.A.); sedakat.altinpinar@gmail.com (S.A.); 2 Deutsches Textilforschungszentrum Nord-West gGmbH, Adlerstr. 1, 47798 Krefeld, Germany

**Keywords:** polymer thin films, roughness correlation, polymer brushes, X-ray scattering

## Abstract

Thin polymer films and coatings are used to tailor the properties of surfaces in various applications such as protection against corrosion, biochemical functionalities or electronic resistors. Polymer brushes are a certain kind of thin polymer films, where polymer chains are covalently grafted to a substrate and straighten up to form a brush structure. Here we report on differences and similarities between polymer brushes and spin-coated polymer films from polystyrene and polymethyl methacrylate with special emphasis on surface roughness and roughness correlation. The phenomenon of roughness correlation or conformality describes the replication of the roughness profile from the substrate surface to the polymer surface. It is of high interest for polymer physics of brush layers as well as applications, in which a homogeneous polymer layer thickness is required. We demonstrate that spin-coated films as well as polymer brushes show roughness correlation, but in contrast to spin-coated films, the correlation in brushes is stable to solvent vapor annealing. Roughness correlation is therefore an intrinsic property of polymer brushes.

## 1. Introduction

**Figure 1:**
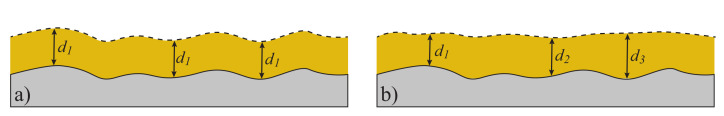
Polymer thin film (yellow) on a silicon substrate (grey) with: (**a**) correlated roughness and therefore a homogeneous layer thickness ($d_{1}$) and (**b**) uncorrelated roughness and different layer thicknesses at three positions ($d_{1}<d_{2}<d_{3})$.

Thin polymer films are of high interest in various applications and disciplines, such as electronics, biomedicals or functional coatings [1,2]. One of the most fundamental properties of such films is the surface roughness [3]. As no surface is ideally flat, height deviations appear, giving the surface a certain structure and roughness profile. Usually, these deviations are described with the Root Mean Square (RMS) roughness, which averages the height deviation from a mean level along a sampling length. A statistical analysis of height irregularities is commonly performed on micro- to nanometer length scales with Atomic Force Microscopy (AFM) or with optical techniques, such as X-Ray Reflectivity (XRR) [3,4,5]. If thin polymer films are coated on a substrate, two roughness profiles exist, namely the silicon-polymer-interface and the top polymer surface. AFM is only useful for measurements of the polymer surface roughness. X-rays however penetrate the polymer layer and therefore XRR also characterizes underlying interfaces, such as the film-substrate-interface [4,5]. Roughness studies of those systems have extensively been performed and reported in literature with the mentioned methods [3,6]. In this report we intend to analyze the phenomenon of roughness correlation of polymer thin films. Depending on the preparation procedure and film thickness the geometries of both interfaces are not necessarily independent from each other. For example in conformal films, the roughness profile from one interface is copied to the overlaying surface, resulting in correlated roughness profiles and consequently a constant layer thickness of the polymer film (Figure 1) [7,8].

**Figure 2:**
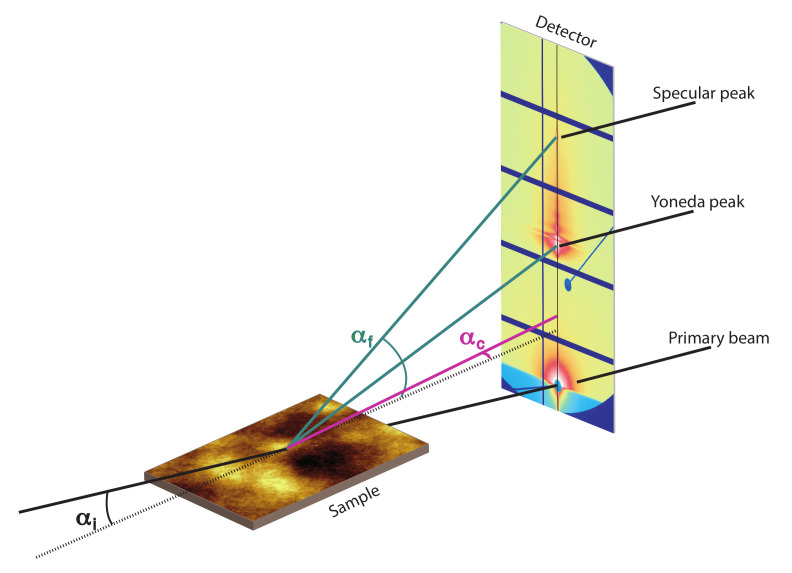
Schematic illustration of a GISAXS setup with X-rays hitting the sample under a grazing incident angle $\alpha_{i}$. The direct beam transmits the sample (primary beam on the 2D-detector), X-rays are reflected (specular peak) and scattered. At $\alpha_{i}+\alpha_{c}$ the so called Yoneda peak appears.

While non-conformal films can be described with an average layer thickness, conformal films show a locally defined layer thickness. If two interfaces are correlated, scattered X-rays of both interfaces undergo constructive interference and additional oscillations (intensity maxima) appear. These signals can theoretically be characterized with XRR, but the intensity is in phase with the oscillations of the reflectivity curve [9,10,11]. Therefore, a simple modeling of XRR curves using a Parratt formalism will lead to erroneous results, especially by modeling interfacial roughnesses, which are systematically too low. In addition to XRR experiments another technique, like Grazing Incidence Small Angle X-ray Scattering (GISAXS) is required, to verify if two interfaces are correlated [8,12,13]. GISAXS is a powerful tool for structural characterization of soft-matter thin films. In GISAXS experiments a 2D detector allows the study of off-specular and non-specular scattering in reciprocal space, so that lateral structures (y-direction) of thin films and structures perpendicular to the substrate surface (z-direction) can be explored in respect to the X-ray beam (x-direction). Coordinates in y- and z-direction in real space correspond to wave vector coordinates $q_{y}$ and $q_{z}$ in reciprocal space. Unlike specular XRR measurements, where the incident angle changes during the measurement, GISAXS commonly may be carried out at a constant incident angle, which is close to the critical angle of the material, to balance a defined penetration of X-rays into the sample and get optimal scattering intensity [12,13]. A schematic illustration of a GISAXS setup is shown in Figure 2.

In a prototypical GISAXS detector image three intensity maxima are observed: The direct beam, which penetrates through the sample, the specular peak, related to the total reflection of the beam and in between a third scattering effect, at an angle equal to the sum of the incident angle and the critical angle ($\alpha_{i}+\alpha_{c}$). This third peak is called Yoneda peak [14]. In correlated films, periodical oscillations in $q_{z}$-direction occur in the scattering images between the specular and Yoneda peak, due to lateral correlations of the scattered X-rays from the substrate/polymer interface and the free polymer surface. Whereas uncorrelated films do not show any roughness related signals between Yoneda region and specular peak. This phenomenon of roughness correlation has been shown with GISAXS for liquid films on substrates by Tidswell and for spin-coated polymer thin films by Gutmann, Stamm and Müller-Buschbaum [7,15,16,17,18,19].

**Figure 3:**
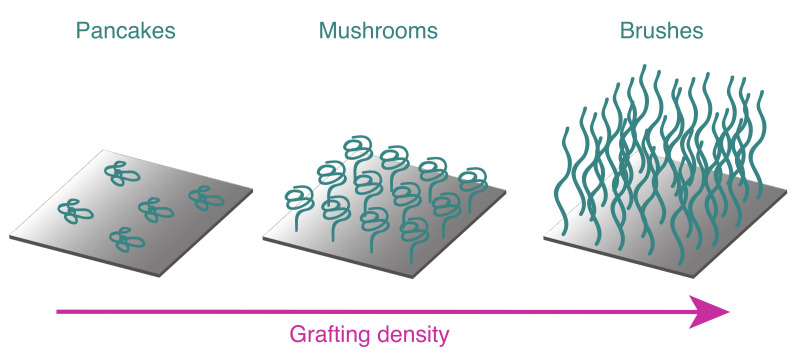
Schematic illustration of the structure of grafted polymer chains with increasing grafting density, from ’pancakes’ via ’mushrooms’ to brushes.

Here we demonstrate roughness correlation of spin-coated polymer films, as well as polymer brushes and show that roughness correlation is an intrinsic property of brush systems. Polymer brushes with correlated roughness to the substrate (conformal brushes) are promising polymer films, for applications, in which stable and homogeneous polymer layers with a locally defined thickness are required, for example organic light emitting diodes. In contrast to spin-coated films, polymer brushes are covalently bond to the substrate surface. Different internal structures of grafted polymers have theoretically been described by Alexander and De Gennes [20,21,22,23,24]. A low number of chains per surface area (grafting density) results in a ‘pancake’ structure, as no interaction between the chains occur. With a higher density of chains within a certain area, repulsive forces become important and the chains straighten up from the surface, losing entropy. ‘Mushroom’ and brush structures are consequently obtained at high grafting densities (Figure 3) [25,26,27]. In polymer brushes the chains are highly stretched and the layer thickness is therefore directly proportional to their molecular weight. For highly uniform brush systems this implies a direct interfacial correlation and enables an investigation of structural transitions in a brush layer via GISAXS experiments.

In this paper the synthesis of polymer brushes is based on a grafting-from approach, in which an initiator for controlled radical polymerization is immobilized on a silicon substrate as Self-Assembled Monolayer (SAM). With Surface Initiated-Atom Transfer Radical Polymerization (SI-ATRP) polymer brushes are synthesized directly on the surface with low polydispersities and high grafting densities [28]. Silicon substrates are used, as they show low surfaces roughness and have extensively been studied with respect to the synthesis of brushes [1,2].

Analogous to the spin-coated films, polymer brushes also exhibit roughness correlation, which has been indicated by Akgun et al. and Kim et al. [29,30]. In this report we compare the surface roughness and roughness correlation of spin-coated polymer films and polymer brushes on silicon substrates, using AFM, XRR and GISAXS as methods for characterization. While AFM and XRR are basically used to characterize the surface structures, RMS roughness and layer thicknesses of all films, GISAXS is the only method to prove roughness correlation from non-specular scattering effects.

## 2. Materials and Methods

Acetonitrile (ACN, VWR), 3-aminopropyldimethylethoxysilane (APDMES) (abcr, 97%), α-bromoisobutyryl bromide (BIBB) (Sigma Aldrich, 98%), CuBr_2_ (Sigma-Aldrich, >99.9%), CuBr (Sigma-Aldrich, >99.9%), dichlormethane (DCM) (VWR), 4-(dimethylamino)-pyridin (DMAP) (Sigma Aldrich), ethanol (Sigma Aldrich), hydrogen peroxide (Carl Roth, 30%), N,N,N′,N′′,N′′′-pentamethyldiethylenetriamine (PMDETA) (Sigma Aldrich, 99%), styrene (Sigma Aldrich, 99%), methyl methacrylate (MMA) (Sigma Aldrich, 99%), water, methanol (Sigma Aldrich), sodium ascorbate (Sigma Aldrich), sulfuric acid (Fischer chemicals, 95%), triethylamine (TEA) (Sigma Aldrich, 99%), inhibitor remover resin (Alfa Aesar), aluminum oxide active basic (Sigma Aldrich). All compounds were used without further purification. To remove the inhibitor hydroquinone monomethyl ether (MEHQ) from MMA and 4-tert-butylcatechol from styrene, MMA was stirred with inhibitor remover resin for 30 min and styrene was passed through a basic aluminum oxide packed column. Silicon substrates were purchased from Active Business Company GmbH Brunnthal Germany.

### 2.1. Immobilization of APDMES on Silicon Wafer

A silicon wafer disk was cut into samples of 2×2 cm${}^{2}$, immersed in ethanol and placed in an ultrasonic bath for 20 min. Afterwards every wafer was thoroughly rinsed with filtered Mili-Q-water and placed in a solution of H_2_SO_4_, H_2_O_2_, and Mili-Q-water in ratio of 15:5:3 for 20 min, before being rinsed with water again. By this procedure, the wafers were activated with hydroxyl groups. The immobilization of APDMES was performed via vapor deposition. After drying with argon, the samples were placed next to a small vial, containing APDMES in a vacuum oven, which was evaporated for 2 h. The vial was removed and the oven was heated up to 110 ${}^{{}^{\circ}}$C for another 2 h, to achieve a complete covalent bond of APDMES with the hydroxyl groups on the silicon surface. Redundant APDMES was removed from the surface by extraction with DCM in a Soxhlet extractor.

### 2.2. Synthesis of 2-Bromo-2-methyl-N-[3-(dimethylsilylethoxy)propyl] on Silicon Substrates

In a round-bottom-flask with magnetic stir bar, DMAP (15.6 mg, 0.13 mmol) was dissolved in 20 mL acetonitrile, while purging the solution with argon. A few minutes later BIBB (247 μL, 1.61 mmol) and TEA were added and stirred for 10 min. While stirring, every APDMES-functionalized wafer was placed in a screw-top vial with a septum and deoxygenated with an argon stream. To each vial a few milliliters of the solution were added, to cover the surface with the liquid. All vials were shaken on a shaker for 3 h, before the wafers were removed and rinsed with ACN and extracted with DCM in a Soxhlet extractor.

### 2.3. Synthesis of PMMA Brushes

Polymethyl methacrylate (PMMA) brushes were synthesized via grafting from approach and ARGET ATRP on functionalized wafers. In a two-neck-round-bottom-flask with magnetic stir bar, 16 mL water and 8 mL methanol were purged with argon for 15 min, before adding inhibitor-free MMA (20 mL, 0.19 mol). The catalyst CuBr_2_ (7.4 mg, 0.03 mmol) with PMDETA (100 μL, 0.48 mol) as ligand and sodium ascorbate (65.3 mg, 0.33 mol) as reducing agent were added. Once all reactants were dissolved, the solution was poured over the functionalized wafers in an oxygen free vial, sealed with a rubber septum. After 20 min the polymerization was stopped and all samples were cleaned with DCM in Soxhlet extractor. The grafting density of 0.93 nm${}^{-2}$ of PMMA brushes was estimated via dry layer thickness approach with the ellipsometric layer thickness and molecular weight of PMMA, which was polymerized in the same solution with sacrificial initiator and analyzed with GPC.

### 2.4. Synthesis of PS Brushes and PMMA-b-PS Brushes

The synthesis of PS brushes was done in a Schlenk tube, containing the functionalized wafers. To assure oxygen free conditions the tube was sealed and purged with argon for 20 min. Anisole (10 mL, 0.09 mol) and styrene (10 mL, 0.09 mol) were added to another tube under argon counter flow and further flushed with inert gas for 15 min. CuBr (13.4 mg, 0.09 mol) was dissolved and the catalytic copper complex was formed with PMDETA (19.6 μL, 0,09 mmol). After dissolving the catalyst completely, three freeze-pump-thaw cycles were performed and the solution was added to the Schlenk tube containing the wafers. While stirring the solution was heated up to 30 ${}^{{}^{\circ}}$C an let react overnight. After polymerization, all samples were purified with DCM.

PMMA-*b*-PS copolymer brushes were synthesized by simply following the two procedures mentioned before but performing the synthesis for the PS part immediately after the cleaning of PMMA brushes with DCM. After ARGET ATRP of MMA, the brushes still have active bromide end groups, which can be used to polymerize styrene. The number of ATRP active PMMA chains per surface area could not be analyzed. With our procedure to synthesize PMMA brushes we were able to prepare brushes with different layer thicknesses up to 150 nm, even with stopping and re-initiating the polymerization. Therefore, we assume that all PMMA chains still have active bromide end groups, which work as immobilized macro initiator for the polymerization of PS for PMMA-*b*-PS diblock copolymer brushes.

### 2.5. Spin-Coating Procedure for Polymer Thin Films

The spin-coating procedure was adapted from Schubert et al., using PS (10 g/L, M${}_{n}$ = 130,520 g/mol) and toluene as solvent [31]. A wafer was adjusted in the sample holder of the spin-coater (Laurell, model WS-650MZ-23npp) and with a syringe 100 μL of the polymer solution were flushed rapidly on the sample, while spinning at 2000 rpm. After another 30 s spinning at the same speed, solvent vapor annealing was done for a few samples. For annealing of the spin coated films, a vial with THF was placed next to the samples in a sealed container for 24 h.

### 2.6. Film Thickness Determination with Ellipsometry

The dry layer thickness of every polymer film was measured with an Optrel multiscope ellipsometer using a wavelength of 632.8 nm at an incident angle of 60${}^{{}^{\circ}}$. To simulate a sample layer model, refractive indices of 3.885 for Si, 1.461 for SiO${}_{2}$, 1.49 for PMMA and 1.5916 for PS were assumed.

### 2.7. AFM Characterization

All AFM measurements were performed with an Agilent Technologies 5500 SPM device in tapping mode. Cantilevers by Micromesh, type HQ:NSC14/AL BS were used. Images were recorded at a scanning speed of 0.5 ln/s with 2048 ln within a 3 × 3 μm${}^{2}$ area. Image processing and analysis were done using the Gwyddion software, version 2.57, to calculate the RMS roughness and the radially averaged Power Spectral Density (PSD) over the whole imaged area.

### 2.8. XRR Measurements

XRR measurements were performed at a Bruker D8 device at the Forschungszentrum Jülich with a Cu-anode lab source and a wavelength of 1.54 Å. Every sample was measured for 2 h, in which the incident angle is changed from 0 to 3${}^{{}^{\circ}}$ within 2 min. The vertical beam size was 0.2 mm. Further processing and analysis were done with Parratt32 software, which is based on Parratt algorithm, to determine layer thickness and RMS roughness [32].

### 2.9. GISAXS Measurements

GISAXS experiments in Figures 11 and 13 were performed at Forschungszentrum Jülich at the GALAXI beamline with a Ga Metaljet source (Kα radiation, E = 9243 eV photon energy, wavelength 0.74 Å), a sample detector distance of 3530 mm and an incident angle of 0.7${}^{{}^{\circ}}$. A Pilatus 1M 2D detector was used with a fully evacuated flight path from source to detector. To compensate for gaps between detector modules in the detector images and line cuts, the detector was moved five times after 12 min of irradiation for each position at a total exposure time of 1 h. Further information about the beamline can be found in literature [33]. All measurements were performed at an incident angle higher than the critical angles of studied polymers ($\alpha_{i}$ = 0.7${}^{{}^{\circ}}$), where the critical angle of PMMA at this wavelength is much lower ($\alpha_{c}$ = 0.14${}^{{}^{\circ}}$). With this setup the beam penetrates the whole sample depths, from the top polymer surface to the substrate-polymer-interface, giving a large separation of Yoneda peak and specular peak on the detector.

Further GISAXS experiments (Figure 12) were performed at Xenoxs Xeuss 3.0 lab system with a Cu-anode (1.54 Å). The sample detector distance was 1100 mm and the incident angle $\alpha_{i}$ = 0.7${}^{{}^{\circ}}$ with 1 h exposure time.

## 3. Results and Discussion

**Figure 4:**
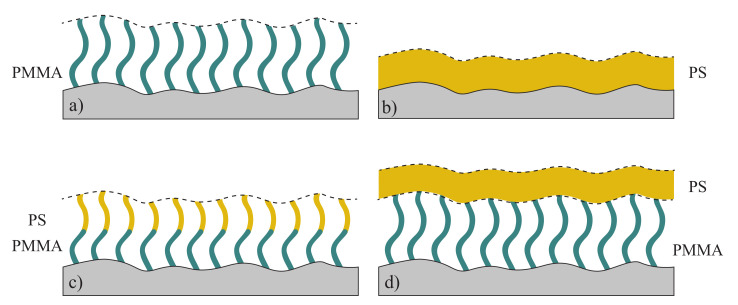
Schematic overview of samples, prepared and characterized in this work. All polymer films were coated on Si-substrates. The dashed lines illustrate the roughness profiles of polymer films, which are correlated to the profile of the substrate. (**a**) PMMA brushes on a silicon substrate; (**b**) Spin-coated PS film; (**c**) PMMA-*b*-PS copolymer brushes; (**d**) PS film spin-coated on PMMA polymer brushes.

The phenomenon of roughness correlation has been shown for liquid films on surfaces, spin-coated thin films and polymer blends. For polymer brushes it has been hinted at. In their studies on spin-coated PS films, Müller-Buschbaum et al. observed a correlated film after spin-coating, which lost all correlations after heating above the glass transition temperature [16,17,18]. During this annealing, temperature increases the mobility of the polymer and the film relaxes from a nonequilibrium state after spin-coating, to an equilibrium stable state. In contrast to spin-coated films, the roughness correlation present in polymer brushes is stable to annealing at high temperatures, which was shown by unpublished results from Ochsmann and Akgun et al. [29,34]. However, a detailed analysis and comparison between both polymer systems has not been published yet. Therefore we concentrate on the similarities and differences in topography, surface roughness and roughness correlation between spin-coated films and polymer brushes and their behavior with respect to annealing. Various samples were prepared, which are shown in Figure 4. As PMMA and PS are standard polymers, which have also been extensively studied in brush systems, both were chosen as materials for these experiments. Next to spin-coated PS films and PMMA- and PMMA-*b*-PS brushes, PS was also spin-coated on top of PMMA brushes, to investigate the roughness correlation of polymer multilayers and prove the persistence of correlated roughness in polymer brushes.

Furthermore, samples with a spin-coated film were characterized with and without solvent vapor annealing. An annealing of polymer brushes was not carried out, as the brushes have been cleaned with boiling DCM in Soxhlet extractor, harsher conditions than vapor annealing with THF. Consequently, if polymer brushes show roughness correlation, this property is seen as intrinsic and stable with respect to annealing.

### 3.1. AFM Analysis of the Surface Structure

As commonly used method to characterize polymer thin films, AFM measurements were performed for an untreated silicon wafer and every sample from Figure 4. With these measurements we can examine differences between spin-coated films and brushes in surface structure and show that roughness correlation can not be verified via AFM. Furthermore, ellipsometry results give information about the layer thickness, but also about the type of polymer, as PMMA and PS have different refractive indices. In contrast to spin-coated films, polymer brushes can not be washed off from silicon substrates with solvents, due to their covalent bond to the silicon surface. We can therefore distinguish between both polymers and verify the successful synthesis of polymer brushes.

**Figure 5:**
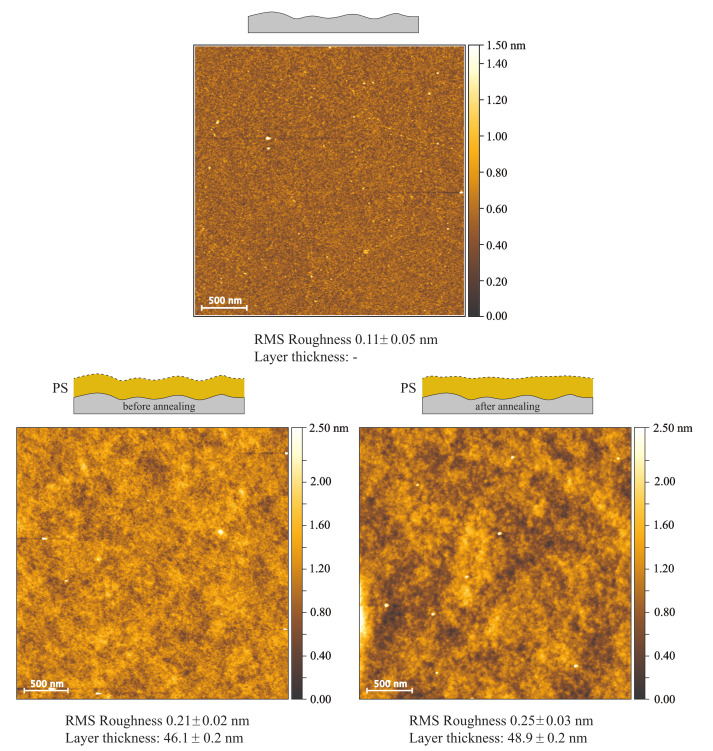
AFM topography images of a bare silicon surface, a spin-coated PS film without solvent vapor annealing and with annealing. Layer thicknesses were measured via ellipsometry and RMS roughnesses of topography images were determined with Gwyddion software.

Figure 5 shows that in comparison to a bare substrate, the RMS roughness of spin-coated films with and without annealing is not significantly increased, only 0.21 nm and 0.25 nm, compared to 0.11 nm for the bare substrate. The ellipsometric layer thicknesses of both films (46.1 nm and 48.9 nm) differs by 2.8 nm, which is a deviation from the spin-coating process and cannot be related to the annealing. Comparing the topography images obtained from AFM measurements of PS spin-coated films with and without annealing, very small changes can be observed, as the lateral size and depth of deeper areas increases with annealing.

**Figure 6:**
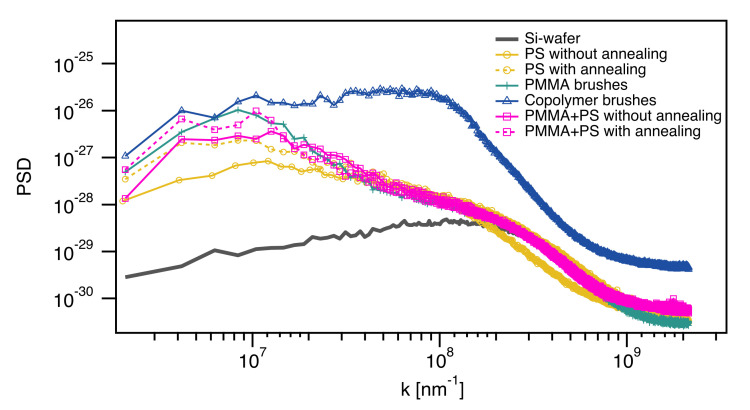
Radially averaged power spectral density of polymer thin films in comparison. PSDs were calculated from AFM topography measurements.

In comparison to the bare substrates an additional lateral structure emerges in the spin-coated systems. This structure manifests in a waviness and is already present before annealing. The associated lateral length is not represented in the RMS values. In order to analyze this length qualitatively we calculate a radially averaged power spectral density (PSD, Figure 6) from the AFM images. In the PSD only minor differences between samples with and without annealing are visible, but both curves exhibit maxima at larger length scales, additionally to the same peak position for smaller length scales, as the blank wafer. A change of distances between higher domains in the topography is also shown by the peak shift of the PSD to larger length scales.

**Figure 7:**
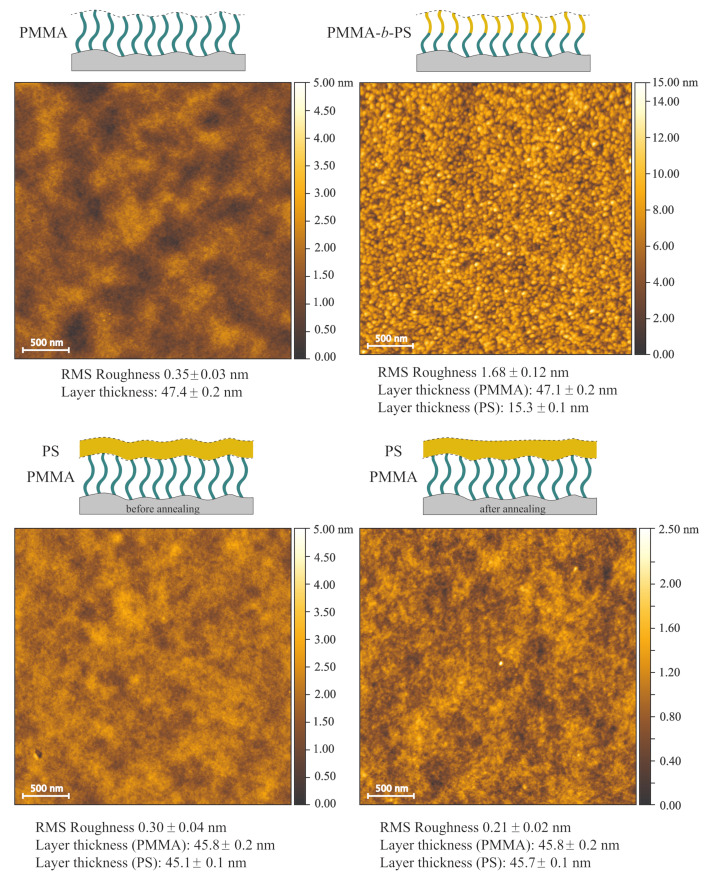
AFM topography images of PMMA brushes, PMMA-*b*-PS copolymer brushes, PMMA brushes with a PS spin-coated film without solvent vapor annealing and with annealing. Layer thicknesses were measured via ellipsometry and RMS roughnesses of topography images were determined with Gwyddion software.

The polymer brush systems also exhibit additional lateral structures. PMMA brushes and spin-coated PS films on top of silicon substrates and PMMA brushes have similar AFM results, regarding topography and RMS roughness (Figure 7). Furthermore PSD functions for these systems are also similar (Figure 6). The dry layer thicknesses of the PMMA brushes and the PMMA layers underneath the spin-coated PS films are between 45.8 nm and 47.4 nm. Due to a low miscibility of both polymers, we assume that no significant diffusion of PS into the brush layer occurs. Therefore two bilayer systems with dry thicknesses of 90.9 nm for the sample without annealing and 91.5 nm for the sample with annealing are obtained.

**Figure 8:**
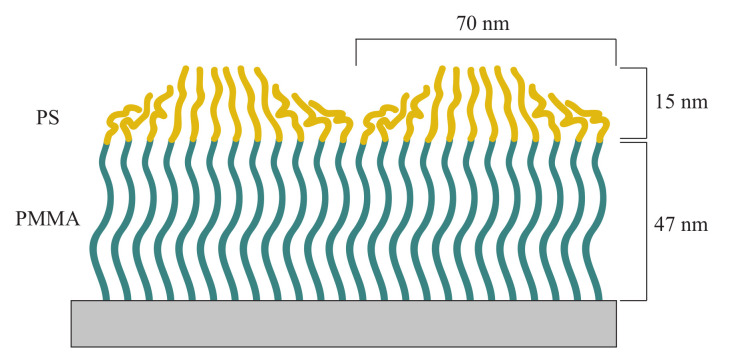
Schematic sketch of PMMA-*b*-PS diblock copolymer brushes in dry state. PS chains accumulate on top of PMMA brushes, to minimize their contact with PMMA and air. This shape is inferred by the layer thickness of 15 nm of the PS block and the PS domain diameter of 69.8 nm.

Diblock copolymer brushes are significantly different in RMS roughness (1.68 nm) and surface topography. In the topography image small domains are visible, caused by PS dimples on top of PMMA brushes, which have a diameter of approximately 70 nm. The miscibility of PS and PMMA is relatively low and the PS blocks will therefore form coils on top of PMMA brushes, to reduce the interaction with PMMA brushes underneath and with air. We assume that the dimples are formed by a few PS chains, which accumulate with stretched chains in the middle surrounded by chains, which bend to the center of the dimple. This behavior of avoidance of diblock copolymer brushes and binary mixed brushes has already been shown in literature and explains the additional maximum in the PSD at a length scale of 70 nm in Figure 6 [35]. A gaussian fit for this peak gives an average diameter of 69.8 ± 2.8 nm for the PS domains. Ellipsometry measurements were performed after synthesis of PMMA brushes and after synthesizing the PS block, in which layer thicknesses of 47.1 nm for PMMA layer and 15.3 nm for PS brushes were recorded. The combination of the diameter of 69.8 ± 2.8 nm and the layer thickness of 15.3 nm (height of the PS domains) indicates an oval shape of the PS domains (Figure 8).

AFM images the surface topography without any information about the underlying interface. Theoretically, a certain position on an uncoated silicon wafer could be measured with AFM and compared with measurements at the same position after coating, but finding the exact same position with an area of interest of 3 × 3 μm${}^{2}$ is impossible. Therefore, X-ray studies are required, enabling the simultaneous analysis of all interfaces and the top surface as well as their correlation. A lateral characterization via AFM in combination with ellipsometric layer thickness measurements is not suited for an investigation of roughness correlation.

### 3.2. XRR Experiments on Polymer Thin Films

**Figure 9:**
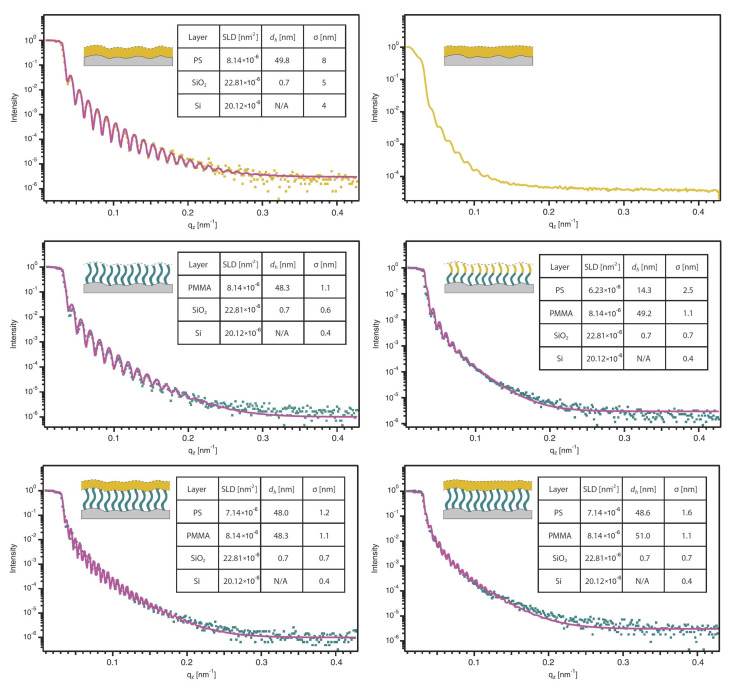
X-ray reflectivity curves of polymer thin films. Measuring points are displayed as dots and fits are presented as solid lines. Note that the measurement for the spin-coated PS film after annealing could not be fitted, as the sample could not be adjusted properly. Via curve fitting procedure, scattering length density (SLD), dry layer thickness ($d_{h}$) and RMS roughness (σ) could be estimated.

From XRR studies of all polymer thin films, RMS roughness and layer thicknesses are determined and compared to AFM measurements. The reflectivity curves in Figure 9 clearly show the expected dependence of 2π/Δq of Kiesig fringes width and layer thickness. While oscillations are clearly observed in spin-coated films on a silicon wafer and on PMMA brushes before annealing, a damping due to increasing roughness is present after annealing.

The PS film on top of the PMMA brushes is expected to be uncorrelated after annealing, while the brush layer is still correlated. Accordingly, two different roughness profiles are stacked in a bilayer system, which explains the increase in RMS roughness after annealing. The X-ray reflectivity curve of the spin-coated PS film after annealing could not be fitted, which may be related to problems in sample alignment and intensity normalization due to an irregular sample shape. Therefore, for the spin-coated PS film only the layer thickness is estimated from the distance between two minima of Kiesig fringes and RMS roughness is not discussed further. For both multilayer samples, two modulations appear with different amplitudes, which is typical for multilayer systems. This confirms our hypothesis from AFM measurements of a multilayered system without diffusion of PS into the brush layer. While XRR results of PMMA brushes are similar to the spin-coated PS film, regarding RMS roughness, the copolymer brushes again show a much higher roughness, caused by the PS domains.

**Figure 10:**
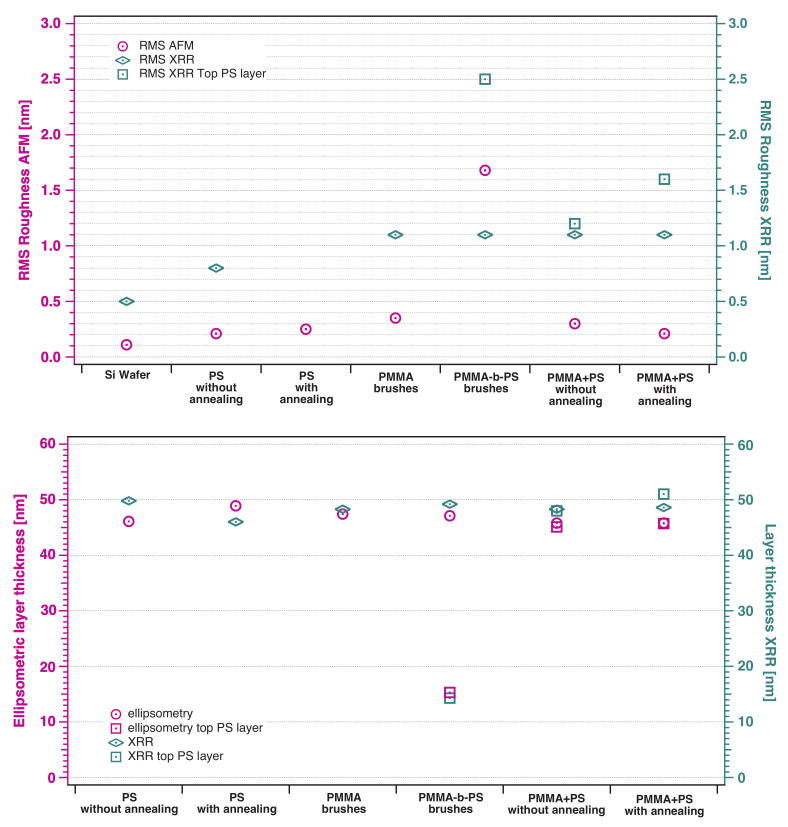
Comparison of layer thickness and RMS roughness results of all samples, which where performed with AFM, ellipsometry and XRR measurements. Top diagram: RMS roughness of a silicon substrate and polymer thin films, measured with AFM and XRR. XRR results are significantly higher than AFM results, which is a hint for roughness correlation. Bottom diagram: Layer thicknesses of polymer thin films, measured with ellipsometry and XRR. Results of both methods are in good agreement.

In Figure 10 the dry thicknesses (measured using ellipsometry and XRR) and RMS roughnesses of all samples (measured with AFM and XRR) are shown. The layer thickness values measured with both methods are in good agreement for all films and differ only by a few nanometers. However, RMS roughness values from XRR are several times higher than values from AFM. One reason for the differences certainly is the area of measurement in both experiments. AFM measurements were taken within an area of 3 × 3 μm${}^{2}$, whereas XRR gives statistics of the sample surface in centimeter-range. The main reason is the correlated roughness. As mentioned in the introduction, the oscillations caused by interference effects of two conformal roughness profiles are in phase with Kiesig fringes, increasing their intensity. At this point the Parratt formalism is no longer valid, as it underestimates the RMS roughness.

If the roughness profiles of substrate and top surface are correlated, non-specular scattering contributes to specular reflection, as both oscillations are in phase, increasing the intensity of modulations. Holy and Baumbach postulated this coherence, considering the distorted wave born approximation in reflectivity measurements [9,10,11]. Thus from the reflectivity curves in Figure 9 only indirect indication for roughness correlations may be obtained. GISAXS results are consequently discussed in the following part.

### 3.3. GISAXS Studies on Polymer Thin Films

**Figure 11:**
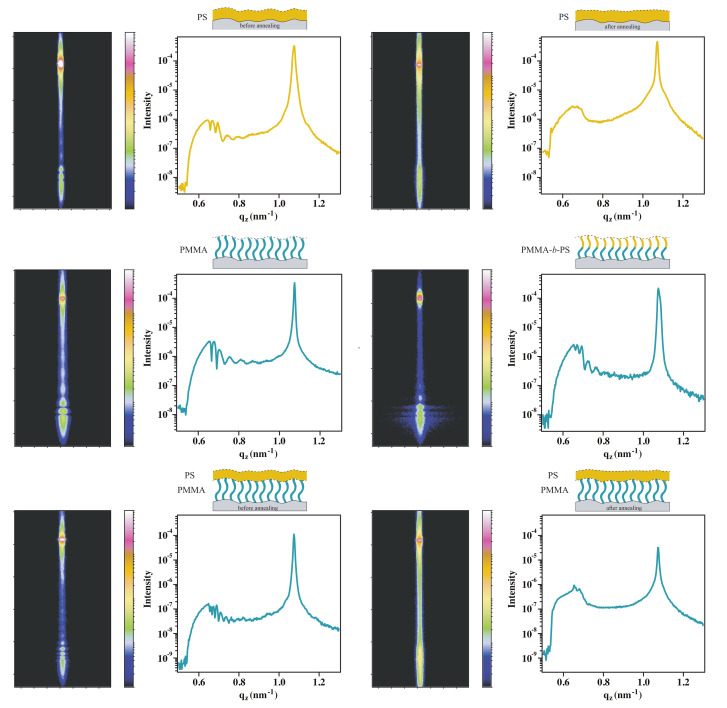
GISAXS detector images and $q_{z}$ detector line cuts of spin-coated PS films with and without annealing, PMMA brushes, PMMA-*b*-PS block-copolymer brushes and spin-coated PS films on top of PMMA brushes.

In the 2D GISAXS measurements of the spin-coated PS films (Figure 11), intensity maxima are observed between Yoneda peak and specular peak [8,15]. With a detector cut (line cut profile) in $q_{z}$-direction, intensity is plotted versus wave vector values, in order to visualize the oscillations more clearly. After solvent vapor annealing, these oscillations disappear, as the roughness is no longer correlated and the scattering of both interfaces are independent. Müller-Buschbaum and Stamm attributed the loss of correlation to a relaxation of the polymer film into an equilibrium state [19]. Here annealing was done via evaporated solvents instead of heating above glass transition temperature, but the effect on roughness correlation is identical. In Figure 11 detector images and line cuts of polymer brush systems are shown, in which intensity oscillations are also present.

Compared to spin-coated films, polymer brushes show a persistent roughness correlation, which is stable to any annealing process. Other polymers than PMMA show the same behavior, as to be seen in the results for PMMA-*b*-PS brushes and for homopolymer PS brushes (Supporting Information). Roughness correlation is therefore an intrinsic property of polymer brushes, independent from the type of monomer.

As further evidence that oscillations in detector cuts are directly linked to roughness correlation and layer thickness of the polymer layer, different layer thicknesses of PMMA brushes were analyzed with ellipsometry and GISAXS. These scattering experiments were conducted at a Xenocs Xeuss 3.0 system.

**Figure 12:**
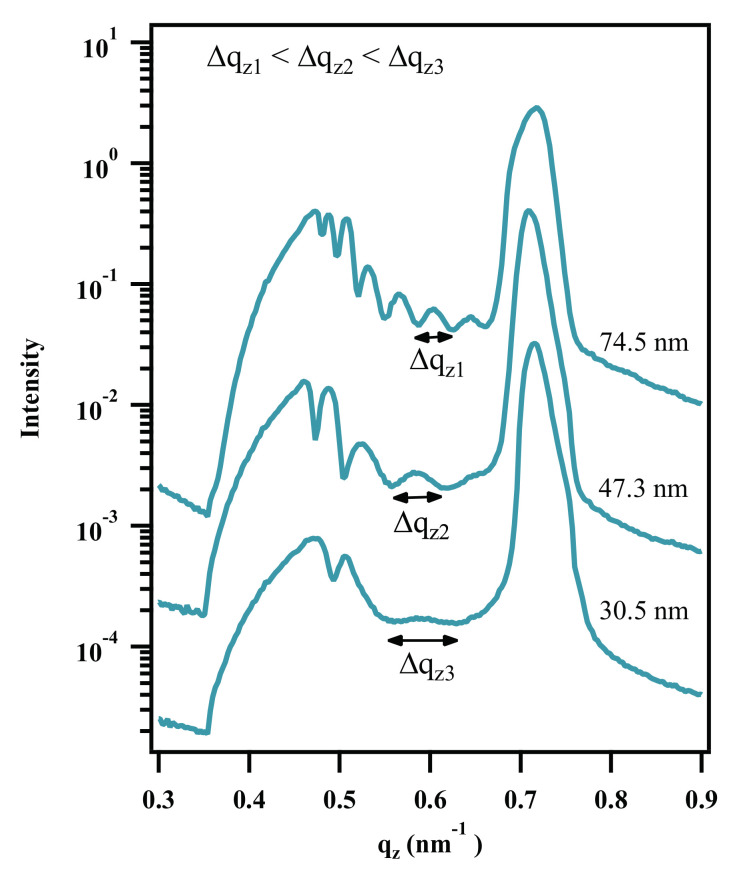
GISAXS line cuts in $q_{z}$-direction of PMMA brushes with different layer thicknesses. The distance between two oscillation minima in the line profiles of PMMA brushes ($\Delta q_{z}$) increases with decreasing layer thickness.

All detector scans in Figure 12 indicate roughness correlation. The oscillation widths shrink from the 74.8 nm brush layer over 47.3 nm to 30.5 nm and show the expected inversely proportional relation of layer thickness and distance between two minima in the line profiles ($\Delta q_{z}$).

**Figure 13:**
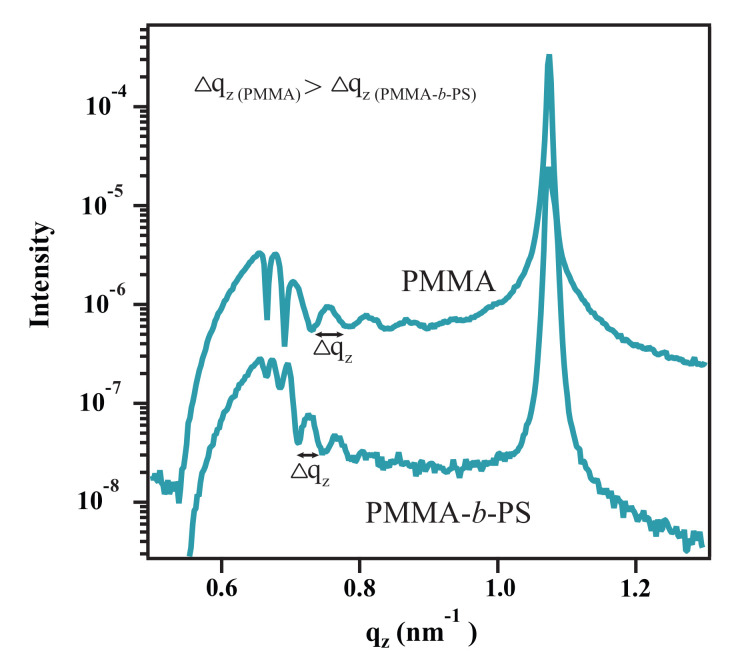
GISAXS line cuts in $q_{z}$-direction of PMMA brushes and PMMA-*b*-PS diblock copolymer brushes. The distance between two oscillation minima in the PMMA brushes ($\Delta q_{z}$) is higher than in copolymer brushes, which is an indicator for a lower layer thickness of PMMA brushes.

**Figure 14:**
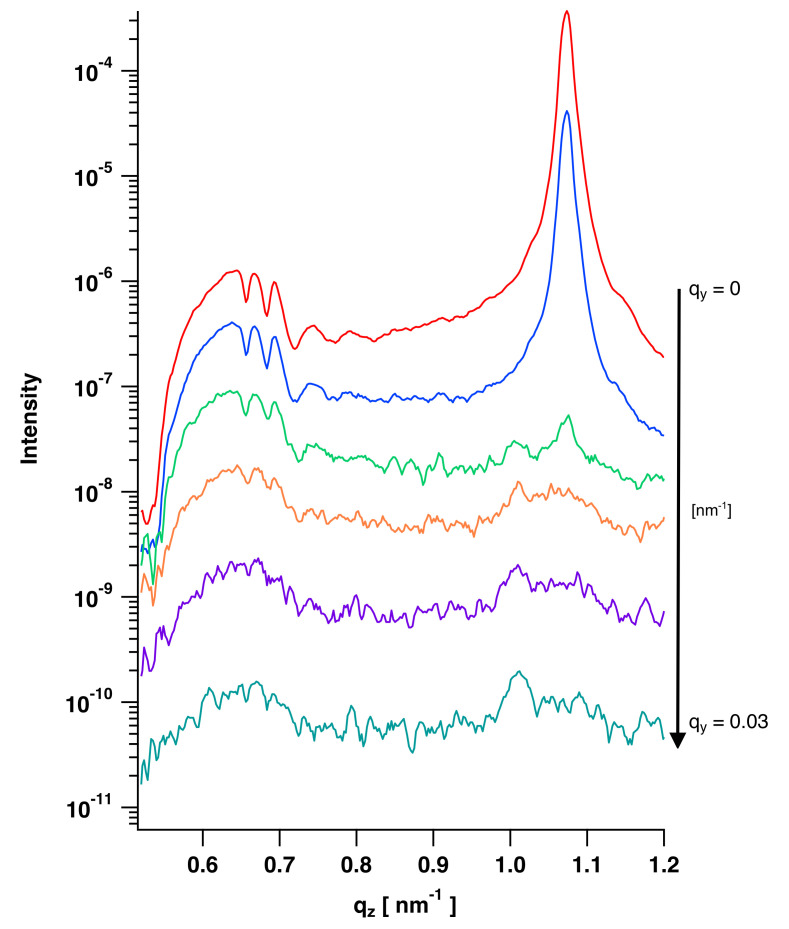
Line cuts in $q_{z}$-direction as a function of $q_{y}$, to determine the lateral cutoff length for roughness correlation of a spin-coated PS film. Intensity is plotted versus $q_{z}$ vector values, starting from $q_{y}=0$ nm${}^{-1}$. At $q_{y}=0.03$ nm${}^{-1}$ modulations are no longer present, roughness is no longer correlated. Note that curves have been shifted for clarity.

Although the block-copolymer brush system shows small domains of collapsed PS chains on top of PMMA blocks, conformality between the surface of the PS domains and the silicon substrate is observed as the width of oscillations in the detector cut matches the thickness of PMMA-*b*-PS brush layer (Figure 13). As an additional proof, that the oscillations are caused by roughness correlation of spin-coated films and brushes, PS films on PMMA brushes are analyzed before and after annealing. Before annealing two different modulations are visible in $q_{z}$-line cut, namely the scattering from the PMMA brushes and PS film (Figure 11). This is in an agreement with our assumption from AFM and XRR results that no diffusion of PS into the brush layer takes place. This assumption is also supported by the Flory-Huggins interaction parameter of PMMA and PS [36,37]. From literature an interaction parameter of

(1) $$\chi =0.028\pm 0.002+\frac{0.39\pm 0.6}{T}$$

is known [38]. Combined with the degree of polymerization *N* for both polymers, the segregation parameter χN can be calculated as 44 for our system. This clearly exceeds the segregation limit of χN≈10 [36,37,39]. Therefore, the existence of conformal polymer multilayers is proven with two different polymer systems, namely brushes and spin-coated films. Again, the oscillations between Yoneda and specular peak disappear after annealing. At $q_{z}$ positions of 0.75 nm${}^{-1}$ and 0.76 nm${}^{-1}$ in the multilayer system with annealing, oscillations are still present related to the PMMA brush layer located under the PS film (Figure 11 bottom right). The conformality of PMMA is therefore independent from the covering PS layer. From these results we can conclude, that polymer brushes, as well as spin-coated films and their multilayer-combinations are able to copy structure information from the substrates to the free film interface. A more detailed view of the lateral extend of these correlations is possible through a more detailed analysis of the lateral cut-off length [15,18]. In correlated polymer thin films, not all structure features of the substrate are replicated to the top surface structure, so that especially small-scale structures get lost during transfer to the upper surface. With the lateral cut-off length $\Lambda_{c}$ the smallest lateral structure size transferred to the top surface can be estimated. To determine the $\Lambda_{c}$ values of correlated films, several $q_{z}$ line cuts along $q_{y}$ are extracted from the 2D scattering image. As a function of increasing $q_{y}$, a decay of modulations along $q_{z}$ is observed. The absolute lateral cutoff length is defined as

(2) $$\Lambda_{c}=\frac{2\pi }{\Delta q_{cor}}$$

where $\Delta q_{cor}$ is the first in-plane wave vector at which all modulations are vanished [15,18]. The pixel position of the beam center is defined as $q_{y}$ value of 0. For a better signal noise ratio, 4 pixels in $q_{y}$-direction are summed up to one line cut. Furthermore, every line cut was smoothed by calculating the median of three values to one value. This procedure gives a clear view on the oscillations. As modulations disappear after annealing, $\Lambda_{c}$ could only be determined for samples without annealing procedure. The position, where the beam center of the direct beam hits the 2D detector is defined as a wave vector of $q_{y}$ = 0 nm${}^{-1}$. Line cuts along $q_{z}$ will therefore be taken at higher pixel values. For example, at a $q_{y}$ wave vector of 0.03 nm${}^{-1}$ modulations of the spin-coated PS film disappear, which corresponds to a lateral cutoff length of 207 nm (Figure 14).

**Figure 15:**
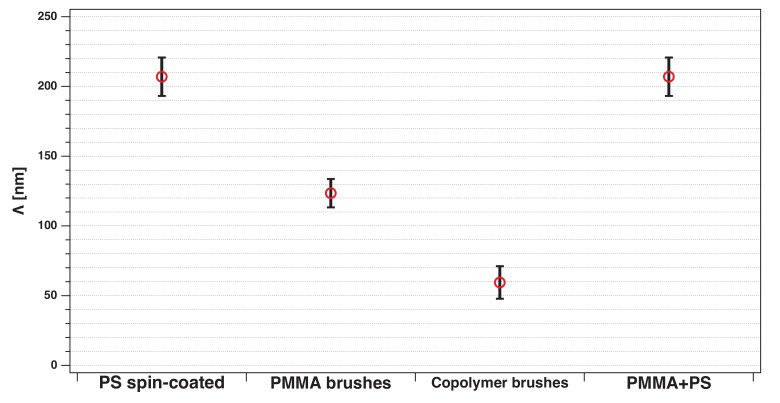
Lateral cutoff lengths for roughness correlation of correlated polymer thin films.

In Figure 15 all $\Lambda_{c}$ values are compared (individual line cuts can be found in he SI). The lateral cut-off lengths for the spin-coated PS film on the substrate and on top of PMMA brushes are equal. Thus the underlying PMMA brush layer seems to behave like a solid substrate, comparable to the silicon wafer. This indicates that in the spin-coated PS films $\Lambda_{c}$ is determined by the hydrodynamics during coating.

For PMMA brushes, the smallest lateral structure length, which is replicated to the polymer surface is around 123 nm. Compared to the spin-coated films, polymer brushes seem to copy smaller length scales of the roughness profile. PMMA-*b*-PS brushes have the lowest lateral cutoff of 60 nm. This is in good agreement with the size of the PS aggregates of 69.8 ± 2.8 nm, indicating that the flat tops of PS dimples (see Figure 8) are also correlated with the conformal PMMA brush surface. From the silicon surface to the top PS layer, especially the larger length scale waviness information get lost.

## 4. Summary

In this report spin-coated polymer films, polymer brushes and multilayers of both polymer systems were analyzed with AFM, ellipsometry, XRR and GISAXS to compare them in regards of surface structure and roughness correlation. We demonstrated the necessity of non-specular scattering experiments to prove roughness correlation of these polymer thin films. While solvent vapor annealing of spin-coated films led to a loss of interfacial correlation, polymer brushes proved stable to solvent annealing processes. We can therefore conclude that roughness correlation is an intrinsic property of polymer brushes.

## Supplementary Materials

The following are available at https://www.mdpi.com/xxx/s1", Figure S1: Detector image and qz line cut of PS brushes, to prove roughness correlation, Figure S2: Determination of the lateral cutoff length c of PMMA brushes (left) and PS brushes (right) via qz line cuts as a function of qy. All curves are shifted for better visibility and represent the mean value of scattering intensities of four pixels with additional smoothing afterwards. Modulations origin from roughness correlation disappear at qy = 0.051 for PMMA and qy = 0.065 for PS, Figure S3: Determination of the lateral cutoff length c of PMMA-b-PS diblock copolymer brushes (left) and PMMA brushes with a spin-coated PS film on top (right) via qz line cuts as a function of qy. All curves are shifted for better visibility and represent the mean value of scattering intensities of four pixels with additional smoothing afterwards. Modulations origin from roughness correlation disappear at qy = 0.106 for copolymer brushes and qy = 0.030 for the PMMA-PS multilayer system.
