# Supplementary material for: Investigation of Roughness Correlation in Polymer Brushes via X-ray Scattering
Source: Polymers (Basel). 2020 Sep 15;12(9):2101. doi: 10.3390/polym12092101 (PMC7569846; doi:10.3390/polym12092101)
Supplement: Supplementary file 1 [file polymers-12-02101-s001.pdf]

Supporting Information

Investigation of Roughness Correlation in Polymer Brushes via X-Ray Scattering

Marcus Hildebrandt [1], Eui-young Shin [1], Suan Yang [1], Wael Ali [2], Sedakat Altinpinar [1] and Jochen S. Gutmann [1,2]

[1] Department of Physical Chemistry and Center of Nanointegration (CENIDE), University of Duisburg-Essen, Universitätsstr. 2, 45141 Essen, Germany

[2] Deutsches Textilforschungszentrum Nord-West gGmbH, Adlerstr. 1, 47798 Krefeld, Germany

GISAXS results of PS brushes

Polystyrene brushes on silicon substrates were synthesized as reported in the corresponding article. The brush layer thickness was measured with ellipsometry, giving a value of 27.1 nm. Roughness correlation of PS brushes was proven with GISAXS as to be seen in the oscillations in 1D q_z line cut (Fig. 1).

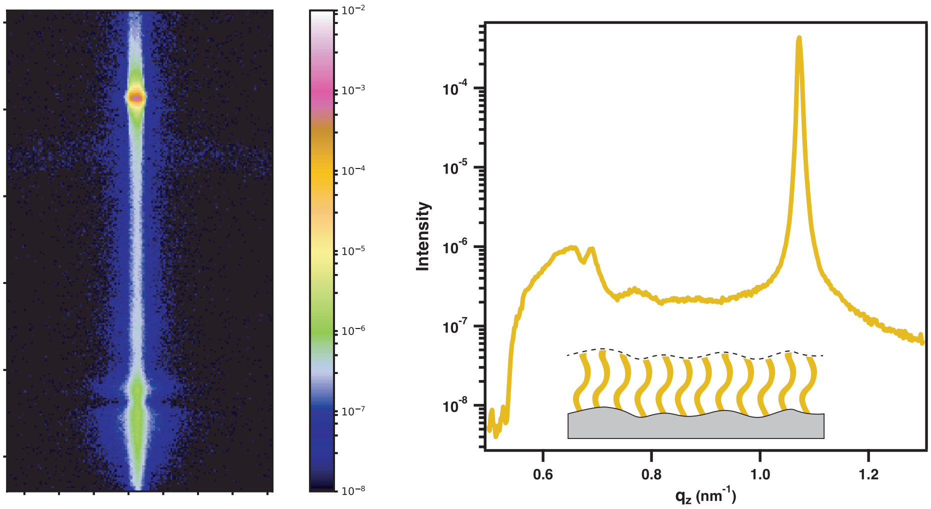

Figure 1: Detector image and q_z line cut of PS brushes, to prove roughness correlation.

Lateral cut-off lengths of polymer thin films

For correlated spin-coated PS films, PMMA brushes, PMMA-*b*-PS brushes and spin-coated PS films on top of PMMA brushes, the lateral cut-off lengths were calculated, using q_z line cuts as a function of q_y.

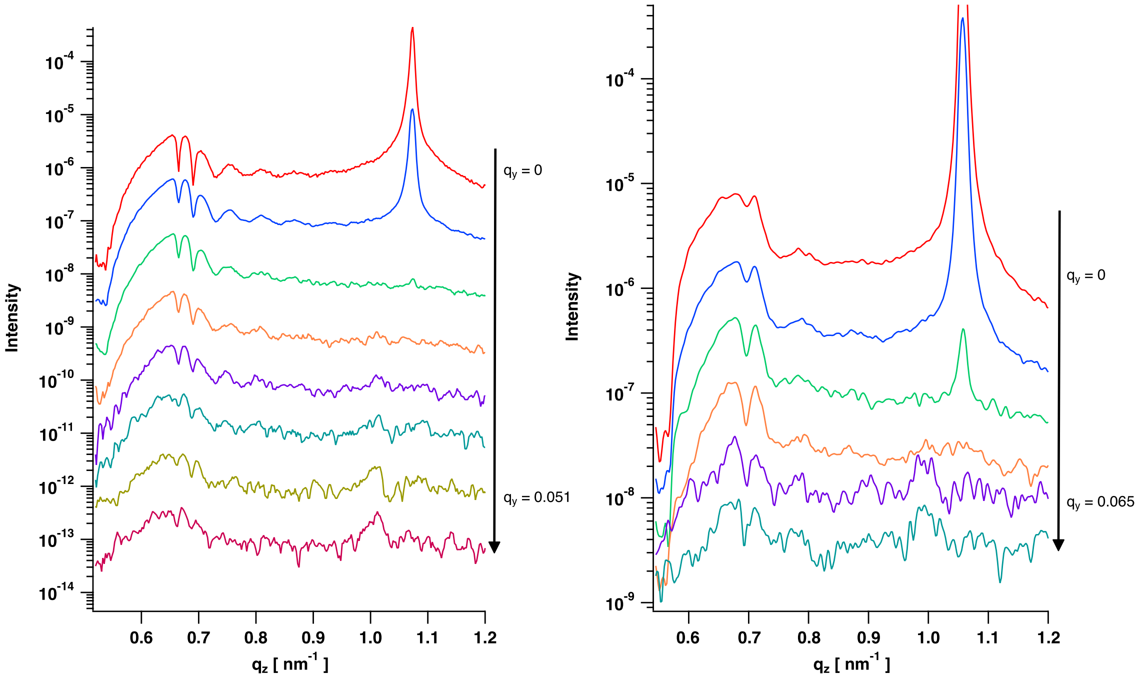

Figure 2: Determination of the lateral cutoff length Λ_c of PMMA brushes (left) and PS brushes (right) via q_z line cuts as a function of q_y. All curves are shifted for better visibility and represent the mean value of scattering intensities of four pixels with additional smoothing afterwards. Modulations origin from roughness correlation disappear at $q_y = 0.051$ for PMMA and $q_y = 0.065$ for PS.

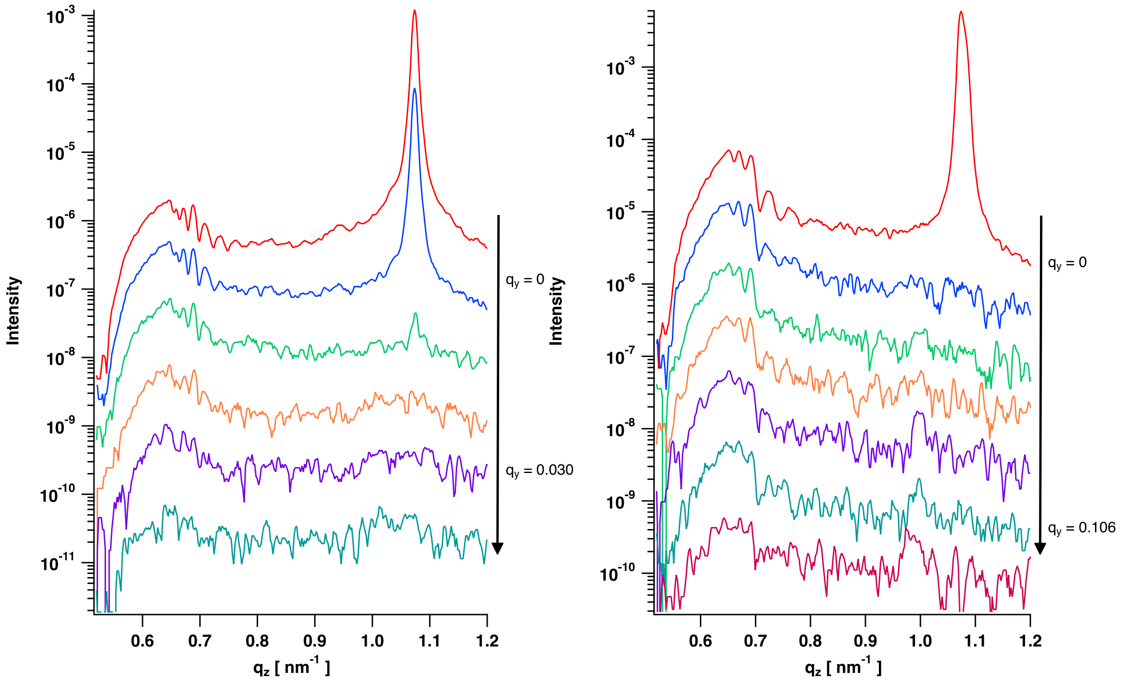

Figure 3: Determination of the lateral cutoff length Λ_c of PMMA-*b*-PS diblock copolymer brushes (left) and PMMA brushes with a spin-coated PS film on top (right) via q_z line cuts as a function of q_y. All curves are shifted for better visibility and represent the mean value of scattering intensities of four pixels with additional smoothing afterwards. Modulations origin from roughness correlation disappear at $q_y = 0.106$ for copolymer brushes and $q_y = 0.030$ for the PMMA-PS multilayer system.
